# End-of-life dementia: demographic, clinical as well as direct health care cost differences in nursing homes vs. ordinary housing

**DOI:** 10.1186/s12913-026-14703-y

**Published:** 2026-05-12

**Authors:** Peter Strang, Dag Salaj, Torbjörn Schultz

**Affiliations:** 1 Department of Oncology-Pathology, Karolinska Institutet, Stockholm, Sweden; 2 Research and Development Department, Stockholms Sjukhem Foundation, Stockholm, Sweden; 3 School of Medical Sciences, Faculty of Medicine and Health, Örebro University, Örebro, Sweden

**Keywords:** Dementia, Palliative care, Healthcare utilization, Healthcare costs, Place of care, Place of death

## Abstract

**Background:**

In late stages, dementia is a palliative diagnosis and the majority of those affected are cared for in nursing homes, but some manage in ordinary housing. The aim was to study how these two groups differ as regards clinical characteristics, healthcare utilization and costs.

**Methods:**

A registry study (Region Stockholm) was performed on deceased patients (≥ 18 years) with a dementia diagnosis during their last year of life, for the years 2015–2023. Pairwise comparisons between nursing homes and ordinary housing were made and multivariable binary logistic regression models were created.

**Results:**

Persons in ordinary housing were significantly younger (84 vs. 86 years old), more often male (51% vs. 39%), they had higher frailty risk scores (Hospital Frailty Risk Scores) and had more often a concomitant metastatic cancer (9% vs. 2%), *p* < 0.0001 in all comparisons. To meet the care needs, these persons received more often specialized palliative care, they had a higher use of geriatric services, they had more emergency room visits and died more often in emergency hospitals, *p* < 0.0001 in all comparisons. In contrast, those who were referred to nursing homes were more often women, older, they had more often Alzheimer´s disease (compared to other dementias), *p* < 0.0001 in all comparisons. As regards medical utilization, the associated costs were equally distributed between women and men in ordinary housing, whereas the costs were significantly higher for men compared with women in nursing homes.

**Conclusions:**

Persons with late-stage dementia in ordinary housing are more often younger, men and they have more often comorbidities, including cancer. They have significantly higher health care utilization, and the costs are equally distributed between women and men.

## Background

The hospice movement, which was the foundation of modern palliative care, initially targeted seriously ill people with cancer, with a clear focus on cancer pain management [1]. Thus, most people with advanced cancer in Sweden still have access to specialized palliative care [2]. Later, neurological diseases such as amyotrophic lateral sclerosis (ALS) [3], as well as diseases with organ failure were also included in specialized palliative care, e.g. heart failure [4] or chronic obstructive pulmonary disease (COPD) [5], but only to a limited extent.

In contrast to these diseases, dementia has not usually been described in terms of a palliative diagnosis, despite the fact that it is a disease with gradual loss of function, increasing symptom burden and often with a fatal outcome. In the age group over 65 years, it is estimated that up to one in three people who die in the United States dies from dementia or with dementia as a strongly contributing cause [6].

Dementia is increasing globally, largely due to increasing life expectancy, which means that people have time to develop dementia: In the age group of 71–79 years, the incidence is estimated to 5%, whereas it is over 30% in those aged 90 and older [7–9]. The disease trajectory differs between primary dementia such as Alzheimer’s disease, with a slow, gradual progression, and secondary, vascular dementia, which deteriorates stepwise with sudden onset of cognitive decline [10]. In addition to Alzheimer’s disease and vascular dementia, there are a large number of other dementia diagnoses. For this reason, dementias early in the disease trajectory constitute a heterogeneous group with many differences, while the functional decline and symptoms are more homogeneous in the final stages of life [10].

Due to slow disease progression over several years, the transition from early dementia characterized by impaired cognitive functions to a terminal, palliative diagnosis is often missed, also by health care staff, despite extensive functional loss and significant symptoms in the final stages of life. In a study, nursing home staff were asked to estimate how many of the newly enrolled residents would survive for six months: According to the staff, only 1% of residents were expected to die within such a limited time frame, but follow-up showed that 71% actually died [11].

However, there are prognostic markers that can be used to support the identification of the transition to a palliative phase with an expected survival of six months on average. For example, the following criteria are often used as Medicare hospice eligibility in the United States: the patient should be on Stage 7 on the Functional Assessment Staging Test (FAST), meaning that the person has significant deficits in motor function [12]. E.g., FAST 7b means that the person only speaks one word clearly, FAST 7e implies “can no longer smile” and 7f means “can no longer hold up head”.

Moreover, they should have at least one of the following complications: aspiration, upper urinary tract infection, sepsis, multiple stage 3–4 ulcers, persistent fever, or weight loss > 10% within 6 months [13]. Considering these losses of functions and complications such as aspiration pneumonias, the burden of care is considerable in the terminal stage. For this reason, a majority of these persons are admitted to nursing homes or similar services, as they need help, assistance and health care around-the-clock, seven days a week. However, in studies exploring preferences for end-of-life (EOL) care, a majority would prefer their own home as the place of death [14] at least when the right conditions are met [15, 16].

When it comes to dementia, caregiving at home often becomes burdensome for loved ones, given that the dying person often needs help with all daily activities and the burden of symptoms is significant. In addition, many people develop behavioral and psychological symptoms of dementia, BPSD, especially in Alzheimer’s disease [17]. BPSD includes many non-cognitive symptoms such as anxiety, depressive symptoms, aggression, disinhibition and sometimes also perception disorders such as hallucinations, which implies great suffering for the person and a significant burden for loved ones [18].

Still, some relatives choose to care for their loved one at home. Stated reasons are caregivers‘ reluctance to send their relative to a care home for emotional reasons, a lack of access to designed palliative care services for people with dementia, as well as a wish to avoid hospital care or other acute care settings [19, 20]. For people who need extensive support with activities of daily life (ADL) at home, the municipality’s home care service is available, with up to eight interventions per day, including night visits. Basic homecare from primary care is available during office hours for those with regular healthcare needs. For people with intense symptoms and complex medical needs, specialized palliative care is an option. Specialized palliative care provides specialized palliative home healthcare around the clock, as well as inpatient palliative care services.

In Sweden, care at nursing homes is provided by the municipality, and the care staff, which mainly consists of assistant nurses and nursing aides, are employed by the municipality, except for physicians, who are employed by the Regions, i.e., the regional healthcare system (formerly known as “county councils”). Licensed nurses with university-level education lead the care at nursing homes and are available during office hours. They are available as on-call nurses during evenings, nights, and weekends. Physicians work on a consultative basis when medical needs arise, but if the need becomes more extensive, temporary hospital care may be required [21].

Thus, the daily basic care and support, including physiotherapy and occupational therapy, at nursing homes are paid for by the municipality. The residents pay rent for their housing at the nursing home, while the regions (in this case, the Stockholm Region) pay for all direct external healthcare costs, which include medical services at the nursing home, medicines, radiology and laboratory tests, etc [22]. If a resident needs additional healthcare, for example, scheduled visits to a specialty clinic at the hospital, unscheduled visits to emergency departments, or unplanned admissions to acute hospitals, the cost is covered by the region. Under special circumstances, specialized palliative home care can also perform individual measures in nursing homes, for example in the case of complex pain problems. In the wider Stockholm Region, there are approximately 14,000 nursing home residents [23].

Most of what we know about palliative care for dementia is based on data from nursing home studies [14, 21, 24, 25], describing financing or care provision models, rather than clinically oriented data [26, 27], or based on studies limited in quantity and certainty [26, 28]. Moreover, there are studies based on data from ordinary housing without comparisons with the conditions in nursing homes [29].

Relatively little has been published about any differences in demographics, clinical characteristics and health care consumption between nursing home residents and those cared for in their own homes. The published studies mainly focus on acute care outcomes and costs [30], they evaluate the determinants of home and nursing home death [31] and assess to what extent palliative care needs are identified and provided in such settings [32]. However, healthcare consumption and, consequently, costs incurred in the care of persons with dementia are associated with variables such as age, sex, frailty, and comorbidities like cancer [21, 22, 31]. Still, surprisingly little has been published on this topic in comparisons between nursing homes and ordinary housing.

### Aims

The aims of this descriptive study were to study possible differences between people with dementia as a main diagnosis in nursing home settings, compared to ordinary housing with regard to demographics (age, sex, type of dementia, frailty risk and cancer as a significant comorbidity), health care consumption and direct medical expenses.

## Materials and methods

### Study design

The manuscript follows The Strengthening the Reporting of Observational Studies in Epidemiology (STROBE) Statement, which are guidelines for reporting observational studies [22]. Data were gathered on all patients who died with dementia 2015 and 2023.

### Population

All patients aged over 18 years who died with dementia as their main diagnosis between 2015 and 2023 in Stockholm county were included, in total 19,244 persons. Those who were registered as nursing home residents when they died were included in the group for nursing home residents, while the others were classified as living in ordinary housing. This classification means that there may be individuals who were residents in nursing homes for a shorter period than the entire last year of life. However, considering that the median length of stay for Swedish residents with dementia is 30 months [33], it indicates that the vast majority in the Nursing Home Residents group lived there throughout their final year.

### Data sources/measurements and variables

The administrative database of Region Stockholm (formerly county council of Stockholm) was used to retrieve data. With the aid of International Classification of Diseases 10th Revision (ICD-10), dementia diagnoses (F00*, F01, F02, F04, G30 and G31.8 A) that have been set by doctors in connection with regular health care visits in outpatient or inpatient settings, were identified.

Dementia was analyzed in relation to the following variables: age, sex, frailty risk as defined by the Hospital Frailty Risk Score [34] and occurrence of advanced cancer, mainly defined as cancer with distant metastases.

The HFRS is an indirect measure of frailty, calculated retrospectively from 109 weighted ICD-10 codes commonly found in administrative data for individuals with frailty. The calculations result in three risk groups: low frailty risk (HRFS < 5), intermediate frailty risk (HFRS 5–15), and high frailty risk (HFRS > 15).

The following outcome variables were used: Receipt of specialized palliative care, which includes specialized palliative home care or inpatient care, basic home care, visits in psychiatric services, emergency room visits, as well as emergency hospitals as places of death.

The difference between specialized palliative care and basic home care is that specialized palliative care is staffed around-the-clock, seven days a week by registered nurses and physicians and daytime also by other professionals, whereas basic home care is run by primary care nurses and general practitioners, mainly daytime and on office hours.

For healthcare cost analysis of direct medical costs, we used Region Stockholm’s simulated costs model (SIM model) for the cost level based on the year 2023. To summarize, the yearly financial statements for each care sector, including somatic specialist care (such as hospital inpatient and outpatient services), geriatrics, psychiatry, primary care, and other care types, were integrated with administrative records from the administrative database. Costs for each documented care event are calculated using various allocation methods, primarily diagnosis-related groups (DRGs, around 50% of expenses) or findings from Swedish National Cost Per Patient projects (covering about 40%), utilizing zero-sum calculations, and actual financial data for the remaining 10%. Since every care event in the database is linked to a specific individual, it is possible to assemble, compute, and compare relative costs across key groups and relevant factors. As described in the Background, costs covered by the municipalities for basic nursing care at the nursing homes are not included in this study: the Stockholm region and the municipalities are separate organizations, and we do not have access to the municipality’s costs.

### Selection bias and dropouts

Reporting to the database is mandatory. Moreover, because every health care intervention must be recorded in the regional database for financial reimbursement purposes, the data set is comprehensive, although occasional human errors in entry can occur.

### Study size

No power calculations were performed as the total cohort (all deaths) was included.

### Statistical methods and missing data

T-tests, Wilcoxon Rank Sum tests, and chi-square tests were applied to compare the groups (pairwise comparisons between nursing homes and ordinary housing).

To analyze which variables were associated with nursing home stays, univariable logistic regression analyses were performed for sex, age groups, type of dementia and the HFRS, which were then entered into multivariable binary logistic regression models if the p-value was below *p* = 0.10. C-statistics were used as the main measure of goodness of fit in our multiple logistic regression model.

Due to the character of data (complete registry data), there was no missing data. The SAS 9.4/Enterprise guide 8.2 was used for the statistical analysis.

## Results

### Demographics and clinical variables (Table 1)

In total, 19,244 persons with dementia as a main diagnosis died between 2015 and 2023, of which 14,717 (76%) resided in nursing homes and 4 517 (24%) in ordinary housing. People in ordinary housing were younger (84.4 vs. 86.2 years, *p* < 0.0001), with a significantly smaller proportion of persons (27% vs. 37%) in the age group of 90 years or older, *p* < 0.0001, Table 1. Also, the sex distribution differed, with more men in ordinary housing, 51% vs. 39%, *p* < 0.0001. The proportion of Alzheimer´s disease versus other dementias did not differ significantly between ordinary housing and nursing home in a univariable comparison, 48% vs. 49%, *p* = 0.32.

**Table 1:** Persons with dementia: demographic and clinical variables

Variable	Total (*n* = 19,244)	In nursing homes (*n* = 14,717)	In ordinary housing (*n* = 4 527)	*p*-value
**Age**,** mean** (SD)	85.7 (7.4)	86.2 (7.3)	84.4 (7.6)	< 0.0001
**Age groups**				< 0.0001
18–64, *n* (%)	160 (1)	90 (1)	70 (2)	
65–79, *n* (%)	3 539 (18)	2 530 (17)	1 009 (22)	
80–89, *n* (%)	8 937 (47)	6 706 (45)	2 231 (49)	
90 or more, *n* (%)	6 608 (34)	5 391 (37)	1 217 (27)	
**Sex**				< 0.0001
Women, *n* (%)	11,177 (58)	8 976 (61)	2 201 (49)	
Men, *n* (%)	8 067 (42)	5 741 (39)	2 326 (51)	
**Type of dementia**				0.32 (ns)
Alzheimer´s disease	9 335 (49)	7 168 (49)	2 167 (48)	
Others	9 909 (51)	7 549 (51)	2 360 (52)	
**HFRS** ^**1**^				< 0.0001
Group 1, *n* (%)	3 108 (16)	2 772 (19)	336 (7)	
Group 2, *n* (%)	8 584 (45)	6 749 (46)	1 835 (41)	
Group 3, *n* (%)	7 552 (39)	5 176 (35)	2 356 (52)	
**Advanced cancer**				< 0.0001
**Yes**	719 (4)	307 (2)	412 (9)	
**No**	18 525 (96)	14,410 (98)	4 115 (91)	

^1^HFRS: Hospital Frailty Risk Score: Group 1 = low risk, Group 2 = intermediate frailty risk, Group 3 = high frailty risk

Persons with high frailty risk (groups 3) as measured by Hospital Frailty Risk Score, was more frequently seen among those in ordinary housing, 52% vs. 35%, *p* < 0.0001, Table 1.

As cancer is known to affect place of care and health care utilization, a separate analysis was performed for people who have both a main diagnosis of dementia and advanced cancer. The proportion of such individuals was much higher in ordinary housing, 9% versus 2%, *p* < 0.0001, Table 1.

### Healthcare utilization (Table 2)

The percentage of people who received specialized palliative care services in the form of specialized palliative home care or inpatient care during the last year of life (LYOL) was 20% for people in ordinary housing and 3% for nursing home residents, *p* < 0.0001, Table 2. Receipt of specialized palliative care was highly correlated with concomitant cancer: in the minority of patients who also had advanced cancer, specialized palliative care services were significantly more common than in those with only a dementia diagnosis. The figures for ordinary housing and nursing homes were 58% vs. 25%, *p* < 0.0001. For patients with dementia but no cancer, the corresponding figures were 16% and 3%, respectively, *p* < 0.0001. Table 2.

**Table 2:** Health care utilization for people residing in nursing homes, versus in ordinary housing

Variable	In nursing homes *n* = 14,717	In ordinary housing *n* = 4 527	*p*-value
SPC^1^ LYOL^2^ Yes/No, *n* (%)	473/14,717 (3)	899/4 527 (20)	< 0.0001
SPC^1^ LYOL^2^*if cancer*, Yes/No, *n* (%)^3^	77/252 (25)	239/412 (58)	< 0.0001
SPC^1^ LYOL^2^*if not cancer*, Yes/No, *n* (%)^3^	396/14,410 (3)	660/4 115 (16)	< 0.0001
Basic home health care LYOL*^2^, Yes/No, *n* (%)	3 632/14,717 (25)	3 017 /4 527 (67)	< 0.0001
Visits in geriatric services LYOL^2^, n (%)	5 786/14,717 (39)	3 540/4 527 (78)	< 0.0001
Visits in psychiatric services LYOL^2^, n (%)	766/14,717 (5)	240/4 527 (5)	0.80
ER visits last month of life, n (%)	4 475/14,717 (30)	2 887/4 527 (64)	< 0.0001
ER visits last month of life, mean (95%CI)^4^	0.4 (0.4–0.4)	0.8 (0.8–0.9)	< 0.0001
Emergency hospitals as place of death	1 576/14,717 (10)	1 423/4 527 (31)	< 0.0001

^1^SPC = specialized palliative care

^2^LYOL = last year of life

^3^In the Table, nursing homes and ordinary housing are compared (rows) with *p* < 0.0001. A within-group comparison between those with and without cancer were also calculated, with significant differences both within nursing homes and in ordinary housing, *p* < 0.0001 in both comparisons

^4^Visits per person (mean value)

Most people in ordinary housing, 67%, received basic home care, the figure for nursing home residents was 25%, *p* < 0.0001. Similarly, the receipt of geriatric services differed, with 78% vs. 39%, *p* < 0.0001, whereas utilization of psychiatric services was low in both groups without differences, Table 2.

There were significant differences as regards emergency room visits: 64% of those in ordinary housing and 30% of nursing home residents made at least one visit during the last month of life, translating to a mean of 0.8 versus 0.4 visits per person, *p* < 0.0001 in both comparisons.

Finally, 31% of people with dementia in ordinary housing had emergency hospitals as their place of death, compared with 10% for nursing home residents (*p* < 0.0001).

### Nursing home residents: a multivariable model (Table 3)

Still, most of the persons with dementia needed the help provided by nursing homes. In our univariable and multivariable regression models, we used sex, age groups, dementia type and HFRS from Table 1 to establish associations. We excluded advanced cancer because such diagnoses were only present in 4% of this population. In our final, multivariable model (Table 3), the same variables that were significant in pairwise comparisons (Table 1) retained their significance, as nursing home residency was strongly correlated with female sex, being older, and having lower frailty risk. Table 3.

**Table 3:** Variables that are associated with a likelihood to be admitted to a nursing home. Univariable and multivariable binary logistic regression analyses

Variable	Univariable		Multivariable^1^	
	OR (95% CI)	*p*-value	aOR (95% CI)	*p*-value
**Sex**				
Women	1.65 (1.54–1.77)	< 0.0001	1.39 (1.30–1.49)	< 0.0001
Men	Ref.		Ref.	
**Age groups**				
18–64 years	Ref.		Ref.	
65–79 years	1.95 (1.42–2.69)	< 0.0001	2.32 (1.67–3.24)	< 0.0001
80–89 years	2.34 (1.71–3.42)	< 0.0001	2.80 (2.02–3.89)	< 0.0001
90 years or more	3.45 (2.51–4.74)	< 0.0001	3.66 (2.64–5.09)	< 0.0001
**Dementia type**				
Alzheimer	1.03 (0.97–1.10)	0.32	1.50 (1.39–1.61)	< 0.0001
All others	Ref.		Ref.	
**HFRS** ^**2**^				
Low risk	3.74 (3.31–4.23)	< 0.0001	4.43 (3.88–5.06)	< 0.0001
Intermediate risk	1.67 (1.55–1.79)	< 0.0001	1.80 (1.67–1.94)	< 0.0001
High risk	Ref.		Ref.	

^1^C-statistic = 0.65 for the full, adjusted model

^2^HFRS: Hospital Frailty Risk Score: Group 1 = low risk, Group 2 = intermediate frailty risk, Group 3 = high frailty risk

Whereas type of dementia was a non-significant variable in unadjusted comparison, an adjusted odds ratio of aOR of 1.50 (1.39–1.61), *p* < 0.0001 was seen in the full model. However, the discriminatory ability of the multivariable model was modest, with C-statistic = 0.65.

### Sensitivity analysis

To ensure that the exclusion of advanced cancer as a variable in the table above (Table 3) does not significantly change the outcome, we performed the same analysis, but excluded all patients who had dementia and concomitant advanced cancer.

All significant results remained and the aORs changed only minimally. The largest single change in aOR was that the HFRS “low risk” group increased from aOR 4.43 in Table 3, to aOR 4.68 when cancer patients were excluded, i.e. negligible changes (data not shown).

### Simulated healthcare costs (Table 4)

Health care costs for the Region (appr. county council) were calculated, whereas costs for the municipalities (including basic costs for nursing homes) were excluded. As the costs were not normally distributed, both median and mean costs per individual were calculated. The median and mean costs in thousand Swedish crowns (tSEK) were 245 and 333 tSEK for those in ordinary housing, compared with 73 and 141 tSEK for nursing home residents, *p* < 0.0001, Table 4.

The median costs for women and men was similar in ordinary housing, 247 and 242 tSEK, respectively, *p* = 0.80, whereas the difference was strongly significant among nursing home residents, 55 vs. 105 tSEK, *p* < 0.0001.

**Table 4:** Health care costs: At the top a comparison between care settings, followed by separate comparisons within each setting, for all variables that are also found in Table 1

Variable	Number (%)	Median (IQR) per individual in SEK 1000	Mean (95% CI) per individual in SEK 1000	*p*-value^1^
**Care setting**				< 0.0001
Ordinary housing	4 527 (24)	245 (133–452)	333 (324–341)	
Nursing homes	14,717 (76)	73 (10–192)	141 (138–144)	
**Sex**, *ordinary housing*				0.80
Women	1 820 (51)	247 (128–462)	336 (324–348)	
Men	1 783 (49)	242 (135–445)	329 (318–341)	
**Sex**, *nursing homes*				< 0.0001
Women	8 976 (61)	55 (8-161)	123 (119–126)	
Men	5 741 (39)	105 (20–235)	170 (164–176)	
**Age groups**, *ordinary housing*				0.36
18–64 years	70	228	318 (247–388)	
65–79 years	1 009	236	338 (320–357)	
80–89 years	2 231	251	336 (324–347)	
90 years or more	1 217	244	323 (308–338)	
**Age groups**, *nursing homes*				< 0.0001
18–64 years	90	55	118 (84–153)	
65–79 years	2 530	80	164 (156–173)	
80–89 years	6 706	86	153 (148–158)	
90 years or more	5 391	54	116 (112–120)	
**Dementia**, *ordinary housing*				< 0.0001
Alzheimer	2 167	231	311 (300–322)	
All others	2 360	259	352 (340–364)	
**Dementia**, *nursing homes*				< 0.0001
Alzheimer	7 168	65	132 (128–136)	
All others	7549	80	150 (145–154)	
**HFRS**^**2**^, *ordinary housing*				< 0.0001
Low risk	336	110	178 (156–201)	
Intermediate risk	1 835	195	277 (265–288)	
High risk	2 356	312	398 (386–410)	
**HFRS**^**2**^, *nursing homes*				< 0.0001
Low risk	2 772	9	33 (30–36)	
Intermediate risk	6 749	51	100 (96–103)	
High risk	5 196	187	252 (246–259)	
**Adv. cancer**^**3**^, *ordinary housing*				< 0.0001
Yes	412	353	433 (405–461)	
No	4 115	235	322 (314–331)	
**Adv. cancer**^**3**^, *nursing homes*				
Yes	307	262	338 (302–373)	< 0.0001
No	14,410	70	137 (134–140)	

^1^Difference (p-value) in median cost per patient between those with non-cancer diagnoses and a diagnosis of advanced cancer using a Wilcoxon rank-sum test

^2^HFRS: Hospital Frailty Risk Score

^3^Adv. cancer refers to patients diagnosed with both dementia and advanced cancer

There were no statistical differences between age groups for those in ordinary housing, whereas the medical care costs in nursing homes for persons aged 65–89 years were higher than for those aged 90 years or more (< 0.0001). In both settings, types of dementia other than Alzheimer’s disease were associated with higher healthcare costs (*p* < 0.0001). In terms of risk of frailty, significantly higher costs were associated with having medium or high frailty risk (*p* < 0.0001). Finally, having a concomitant advanced cancer was also associated with significantly higher health care costs (*p* < 0.0001).

## Discussion

Compared to nursing home residents, people in ordinary housing were younger, more often men, they had a higher frailty risk (HFRS), and they had more often a concomitant, advanced cancer disease. Persons with dementias other than Alzheimer´s disease were more likely to be found in ordinary housing.

In terms of care utilization, people in ordinary housing more frequently received specialized palliative care, particularly those with concomitant cancer, and they also were more likely to access basic home health care services. Moreover, they made significantly more visits in geriatric services, as well as emergency room visits and died more frequently in emergency hospitals. As regards healthcare costs, higher costs were found for those in ordinary housing. Within respective group, women in nursing homes had significantly lower costs than men, whereas no gender difference was found for persons with dementia in ordinary housing.

There were more women with dementia in nursing homes than in ordinary housing, well in agreement with other studies [35–37]. Although the study was not designed to establish causality, it is our belief that women are more likely to provide care for a sick spouse at home, and to a greater extent, than men do for their wives. A possible contributing reason may be that men generally receive more medical interventions than women [37], even in the very end of life, and such interventions are usually provided outside nursing homes. Men with significant medical needs are therefore less likely to be referred to nursing homes. This assumption is partially supported by a study by Baum and colleagues [38], who found that less than a quarter of women who died in nursing homes had spouses, and that two-thirds of the deceased had only adult children as close relatives.

Moreover, even among nursing home residents, men and younger individuals are more likely to be referred to emergency hospitals for acute illnesses [21]. Finally, demography contributes to the understanding because women live longer and therefore many women survive their male spouses.

Age was a significant variable, with a higher mean age in nursing homes, in line with data from a Swedish national study [39]. This was an expected finding, as people prefer to live in their own homes if possible and on a group level, younger individuals are more likely to have younger spouses who can assist with informal care at home.

In the adjusted, multivariable model, persons with dementias other than Alzheimer´s disease were more likely to reside in ordinary housing whereas persons with Alzheimer´s disease resided more frequently in nursing homes. One possibility is that Alzheimer’s disease, as a primary form of dementia, has slower progression, compared to vascular dementias. Moreover, vascular dementia is often a manifestation of other vascular diseases, which may have faster progression and affect other organs, thereby resulting in different needs. Our clinical experience suggests that the prolonged trajectory related to Alzheimer´s disease provides more time for care planning and for a transfer to a nursing home.

Higher frailty risk measured with HFRS was to a greater extent associated with ordinary housing, which we had not expected as frailty is a common indication for admission to a nursing home. This may possibly be explained by the fact that HFRS is actually based on 109 weighted ICD-10 diagnoses that are often seen in frailty [34], i.e. HFRS based frailty risk is actually a measure of comorbidity. Some forms of comorbidity such as cancer may require a higher level of medical expertise than nursing homes can generally offer. In a recent Delphi survey, comorbidities and complications were defined as one of five domains that is associated with a need of specialized palliative care, which is found outside nursing homes [40].

Since cancer can particularly affect where people receive care, we examined those with both dementia and advanced cancer. Our findings showed that a notably larger number of these individuals resided in regular housing. This was an expected finding as persons with advanced cancer, even those with concomitant dementia, may have ongoing cancer treatments in the last year of life. Moreover, they regularly have complex symptoms and needs that are best provided by specialist palliative care. In fact, as many as 79% of patients with advanced cancer receive specialist palliative care in the Stockholm region [2]. In our data, 58% of those in ordinary housing with diagnoses of dementia and cancer received specialist palliative care. This lower figure is likely due to the fact that some people with dementia suffer from severe BPSD, which is best managed in designated dementia wards in nursing homes even in the presence of concurrent cancer. On the other hand, it places increased demands on nursing homes to be able to manage complex cancer symptoms in the final stages of life.

When it comes to people with dementia as their main diagnosis, the criteria to support timely referral to specialist palliative care are less defined. In a systematic review by Mo et al., different indications such as late stage of dementia, e.g., identified with the aid of Functional Assessment Staging Test (FAST), poor prognosis, medical complications including acute infections and falls / fractures were mentioned, but without consensus [41]. In a recent, international Delphi survey, five main categories that indicate need for specialized palliative care were identified, namely (1) dementia type (e.g., rapidly progressive dementia), (2) severe physical symptoms,3. complex psychosocial aspects including request for hastened death, 4. comorbidities or complications such as episodes of aspiration pneumonia and 5. frequent hospital use [40].

Awareness that dementia is a palliative diagnosis in late stages is still low, but awareness can be improved. In a randomized study of hospitalized individuals who received targeted palliative support for dementia, a 60-day follow-up showed that those who received support were more likely to be referred to hospice. Additionally, their families were more frequently offered discussions about prognosis and goals of care [42]. Greater awareness will hopefully translate into better palliative care.

### Home help and health care utilization

Caring for a relative in ordinary housing is a great burden, even with the support available. Home care services assist, for example, with dressing and undressing, meals, toilet visits, showering, and cleaning, which is helpful for relatives.

In case of basic medical needs, such as monitoring chronic heart failure or COPD, a person living in ordinary housing can either receive help through their primary care center, where the general practitioner and district nurse work, or they can access basic home health care, run by the primary care center, if the needs are more frequent. As expected, persons in ordinary housing had significantly greater needs, as basic medical care is already provided in nursing homes, by the staff.

If a person with dementia has extensive and complex medical needs, specialized palliative care can be accessed while in ordinary housing. Ideally, specialist palliative care is provided at home, though inpatient care may also be an option. Such needs of specialized palliative care arise especially if the person has a concurrent symptomatic cancer or another symptomatic organic disease. Therefore, the receipt of specialized palliative care was significantly higher for persons in ordinary housing than in nursing homes, and especially in cases with concomitant advanced cancer.

Due to the Swedish organization of healthcare where nursing homes are funded by the municipality and specialized palliative care by the county council, it is rare for people in nursing homes to have access to specialized palliative care. However, there are limited agreements that enable specialized palliative homecare teams to be consulted by nursing homes, which explains why only a small minority receive specialized palliative care in nursing homes. Moreover, if a nursing home resident has urgent medical needs that cannot be met at the nursing home, the person may be referred to an emergency room, at an emergency hospital. When such referrals from nursing homes to emergency departments are needed, the patients are more likely to be male, younger and have high frailty risk as measured by HFRS [21]. Clearly, the limited medical interventions available in nursing homes without specialized palliative care access are often insufficient to address more complex cases. This may partly explain why those living in ordinary housing were younger, more often men and had higher frailty risk scores.

To be able to die in one’s preferred place of death is often regarded as an indicator of quality [43]. Since numerous studies demonstrate that most people do not wish to die in emergency hospitals, and that dying there often reflects a failure to achieve the patient’s preferred place of death [43, 44], we chose to contrast emergency hospitals as the place of death with all other locations. We found that individuals living in ordinary housing died in emergency hospitals significantly more frequently than nursing home residents, 31% compared to 10%.

At the same time, some criticism has arisen regarding the association between hospital death and poor quality of care [45]. It is now understood that the place of death alone does not fully reflect the quality of care, as a “good death” can also occur in a hospital setting, provided that appropriate care is given.

### Health care costs

For obvious reasons, regular healthcare costs that are financed by the Region (formerly: county council), were higher for those in ordinary housing, as nursing homes are financed by the municipalities. Nursing home residents’ medical costs only arise when the care at the nursing home is not sufficient, and they need to visit an outpatient clinic or need to be admitted to an emergency hospital.

However, when the costs of men and women were compared, interesting differences were seen. The healthcare costs were the similar for men and women in ordinary housing, which at the group level indicates equal care. However, gender-related differences were seen when it came to nursing homes, with significantly higher costs for men, in line with a previous detailed analysis where costs for men and women were compared [22].

### Strengths and limitations

A general strength is the size of the study and a negligible dropout rate.

A limitation is associated with the HFRS: Frailty risk as calculated by the HFRS method, relies on actual ICD-10 codes ICD 10 made by physicians in routine care. Persons in ordinary housing visit primary care or hospital care where diagnoses are set for every visit, whereas nursing home residents are infrequently visited by physicians and there is a greater risk that formal diagnoses are not set. For this reason, the proportion of nursing home residents with high frailty risk is probably underestimated and the reported associations may be partially attributable to measurement bias related to healthcare utilization patterns.

## Conclusions

Persons with late-stage dementia in ordinary housing are more often younger, men and they have higher frailty risk and more often cancer. They have significantly higher health care utilization, and the costs are equally distributed between women and men. Consequently, it is essential to consider demographic variations when comparing healthcare utilization and associated costs between the two settings.

## Data Availability

The dataset contains personally identifiable information, such as personal identity numbers, and date of death, and is therefore subject to ethical and legal restrictions on public sharing, according to Swedish laws. However, datasets generated, used and analyzed during the current study are available from the corresponding author on reasonable request.
